# L’apport des abords vasculaires centraux dans la prise en charge des patients recevant une chimiothérapie pour des tumeurs malignes non hématologiques: PICC lines versus chambres implantables

**DOI:** 10.11604/pamj.2026.54.8.50864

**Published:** 2026-05-12

**Authors:** Ouassila Bendjaballah, Sofiane Chioukh, Assia Bensalem, Hichem Makhloufi, Atika Karoune, Abdelhak Lekhal, Rima Mouellef

**Affiliations:** 1 Service d'Anesthésie-Réanimation, Établissement Hospitalier Didouche Mourad, Constantine, Algérie,; 2 Faculté de Médecine, Université Constantine 3, Constantine, Algérie,; 3 Service des Urgences Médicales, Centre Hospitalo-Universitaire Constantine, Constantine, Algérie,; 4 Service d'Oncologie Médicale, Établissement Hospitalier Didouche Mourad, Constantine, Algérie,; 5 Département d'Anesthésie-Réanimation, Centre Hospitalo-Universitaire Constantine, Constantine, Algérie,; 6 Service d'Épidémiologie et Médecine Préventive, Établissement Hospitalier Didouche Mourad, Constantine, Algérie

**Keywords:** Accès veineux central, Port, PICC, complications, coût, oncologie, Algérie, Central vascular access, Port, PICC, complications, cost, oncology, Algeria

## Abstract

**Introduction:**

les cathéters centraux à insertion périphérique (PICC) et les chambres implantables (Ports) sont utilisés pour la chimiothérapie. Cette étude vise à comparer leur morbi-mortalité et l'incidence des complications.

**Méthodes:**

une étude prospective, comparative randomisée et monocentrique, avec un suivi de 6 mois, a inclus des patients adultes atteints de tumeurs non hématologiques, éligibles à une chimiothérapie (intention palliative, curative ou adjuvante). Les critères d'évaluation principaux étaient la fréquence des événements indésirables (mineurs/majeurs), l'impact sur la qualité de vie (EORTC QLQ-C30) et le coût médico-économique.

**Résultats:**

sur 206 patients randomisés, 192 ont été analysés (Port: 102; PICC: 90). L'incidence globale des complications était de 12,5% (n=24). Les complications majeures étaient significativement plus fréquentes avec les PICC (17,8%; 16/90) qu'avec les Ports (4,9%; 5/102), avec un risque relatif de 3,6 (IC95% 1,3-9,9; p=0,041). Pour une chimiothérapie de moins de 6 mois, le coût total moyen était significativement plus élevé pour les Ports (160 123,41 DA) que pour les PICC (43 259,69 DA) (p<0,001). Aucune différence significative n'a été observée sur la qualité de vie globale, avec une tendance modeste en faveur des Ports pour la santé globale et la douleur à trois mois.

**Conclusion:**

les Ports, bien que plus coûteux, sont associés à un risque significativement plus faible de complications majeures. Ils pourraient être préférés comme accès sûr et efficace pour les chimiothérapies de longue durée. Le choix optimal doit intégrer la durée du traitement, le profil de risque du patient et les contraintes économiques.

## Abstract (en)

**Introduction:**

peripherally inserted central catheters (PICCs) and totally implantable ports (Ports) are used for chemotherapy administration. This study aimed to compare their morbidity, mortality, and complication rates.

**Methods:**

a prospective, randomised, comparative single-centre study with 6 months of follow-up included adult patients with non-hematological malignancies eligible for chemotherapy (palliative, curative, or adjuvant intent). Primary endpoints were the frequency of adverse events (minor/major), impact on quality of life (EORTC QLQ-C30), and medico-economic cost.

**Results:**

out of 206 randomised patients, 192 were analysed (Port: 102; PICC: 90). The overall complication rate was 12.5% (n=24). Major complications were significantly more frequent with PICCs (17.8%; 16/90) than with Ports (4.9%; 5/102), with a relative risk of 3.6 (95% CI 1.3-9.9; p=0.041). For chemotherapy lasting less than 6 months, the mean total cost was significantly higher for Ports (160,123.41 DA) than for PICCs (43,259.69 DA) (p<0.001). No significant difference was observed in overall quality of life, with a modest trend favoring Ports for global health and pain at three months.

**Conclusion:**

Ports, although costlier, are associated with a significantly lower risk of major complications. They may be preferred as a safe and effective access for long-term chemotherapy. The optimal choice should integrate treatment duration, patient risk profile, and economic constraints.

## Introduction

L'usage d'une voie veineuse centrale est toujours nécessaire chez les patients cancéreux, la pose de matériel implantable se justifie dans leur parcours de soins aussi bien dans un objectif thérapeutique de soins curatifs nécessitant des chimiothérapies le plus souvent agressives, que dans un objectif de soins de support avec fréquemment des besoins de nutrition parentérale ou transfusionnels. Ces produits étant agressifs pour le capital veineux superficiel, il est impératif de disposer d'une voie d'abord de débit suffisant. Parmi les différents types de cathéters veineux centraux (CVC), les PICC et les chambres implantables sont les plus couramment employés. Le choix entre ces dispositifs est souvent arbitraire et repose sur une évaluation multidimensionnelle intégrant leur profil de complications, leur impact sur la qualité de vie et leur coût-efficacité.

Les PICC présentent comme avantage initial une mise en place technique simplifiée, évitant ainsi certaines complications mécaniques [1], réalisable en dehors du bloc opératoire, et un investissement financier initial moindre [2]. Cependant, cette apparente praticité doit être tempérée par la constatation d'un risque thrombotique significativement élevé, avec une incidence rapportée de thromboses veineuses profondes près de quatre fois supérieure à celle des Ports [3]. Inversement, les Ports démontrent une supériorité en matière de prévention des complications infectieuses. Les données actuelles indiquent une incidence notablement réduite de bactériémies liées au cathéter avec ce dispositif [4]. Néanmoins, certaines études contredisent ces résultats, ne montrant pas de différence significative dans l'incidence composite des complications entre les deux dispositifs [5].

La dimension de la qualité de vie des patients mérite une considération particulière. Les études comparatives montrent une satisfaction des patients significativement supérieure avec les Ports, particulièrement concernant le confort dans les activités quotidiennes et l'image corporelle [6]. Cependant, d'autres travaux soulignent que cette préférence peut varier selon le profil des patients et leurs activités professionnelles. Les *guidelines* de l *'American Society of Clinical Oncology* (ASCO) et de l'*European Society for Medical Oncology* (ESMO) reconnaissent explicitement l'absence de consensus clair pour déterminer le dispositif le plus sûr, prônant une décision individualisée [7,8]. Face à ces données scientifiques, le choix thérapeutique entre PICC et Port nécessite une approche personnalisée qui intègre le profil de risque du patient, la durée prévue du traitement, l'impact sur la qualité de vie et les considérations économiques. Cette complexité décisionnelle souligne la nécessité de mener des études comparatives supplémentaires afin d'éclairer les pratiques cliniques et d'optimiser la prise en charge des patients en oncologie. Afin de fournir des preuves pour la sélection clinique du dispositif d'accès veineux central optimal, nous avons mené une étude prospective, comparative, randomisée et monocentrique. Cette étude visait à comparer les complications, le coût et la qualité de vie entre les PICCs et les Ports.

## Méthodes

**Conception et cadre de l'étude:** il s'agit d'une étude prospective, comparative, randomisée et monocentrique, menée au sein du service d'anesthésie-réanimation de l'établissement hospitalier universitaire Didouche Mourad (Constantine, Algérie), entre novembre 2022 et octobre 2024.

**Population d'étude:** nous avons inclus des patients adultes (18 ans) atteints de tumeurs solides, candidats à une chimiothérapie avec une espérance de vie estimée supérieure à 4 semaines. Les critères d'exclusion comprenaient les infections systémiques sévères, les thromboses veineuses cliniquement significatives, les antécédents thromboemboliques, l'incapacité à communiquer et la nécessité d'une fistule de dialyse.

**Échantillonnage:** le calcul de la taille d'échantillon a été réalisé par un épidémiologiste, sur la base des données de l'étude suédoise PICCPORT [8], visant une puissance de 90% avec un risque α de 5%, et en supposant un taux de complications thrombotiques de 8% dans le groupe PICC et 1,6% dans le groupe Port, 97 patients ont été nécessaires.

**La randomisation:** la randomisation a été effectuée manuellement selon un ratio 1: 1 en utilisant une table de nombres aléatoires. L'allocation a été dissimulée dans des enveloppes opaques scellées numérotées séquentiellement, ouvertes par la secrétaire du service après inclusion et obtention du consentement éclairé. Les patients ont ainsi été assignés à recevoir soit un PICC, soit une chambre à cathéter implantable Port.

**Interventions:** groupe PICC: pose d'écho guidée au lit du patient par un médecin anesthésiste-réanimateur expérimenté ; groupe Port: pose d'écho guidée au bloc opératoire sous anesthésie locale par un médecin anesthésiste-réanimateur expérimenté.

**Les critères d'évaluation:** le critère principal composite comprenait la survenue de complications (mineures/majeures) thrombotiques, infectieuses, occlusives et mécaniques liées au dispositif nécessitant une intervention.

Les critères secondaires incluaient le succès technique, la durée procédurale, le coût global et la qualité de vie évaluée par le questionnaire (EORTC QLQ-C30) validé en langue française.

**Définitions:** les complications majeures incluent un des critères suivants [9]: la survenue d'un événement de grade 3 et plus selon l'échelle (CTCAE 4.0); un retard de chimiothérapie >7 jours; un changement de dispositif; un événement menaçant le pronostic vital ou nécessitant une hospitalisation. La thrombose: symptômes cliniques confirmés par imagerie (échographie Doppler); l'infection: selon les critères de l'*Infectious Diseases Society of America*; l'occlusion: incapacité à perfuser nécessitant une intervention thrombolytique; complications mécaniques: rupture, migration ou dysfonction du cathéter. Le coût moyen total = le coût moyen de pose (le coût du dispositif est inclus) + le coût moyen d'entretien + le coût moyen des complications + le coût moyen de retrait. Les tarifs des dispositifs et des hospitalisations ont été recueillis via le bureau de la direction des finances et du matériel (DFM).

**La collecte des données:** les patients ont été suivis prospectivement avec des visites programmées à 1 mois, 3 mois et 6 mois après l'insertion du dispositif. À chaque visite, nous avons réalisé un examen clinique complet, administré le questionnaire EORTC QLQ-C30, documenté toute complication et collecté les données économiques.

**L'analyse statistique:** les analyses ont été réalisées avec SPSS version 26. Les comparaisons ont utilisé le test du Chi^2^/Fisher (variables qualitatives) et le test t de Student/ANOVA (variables quantitatives). Une analyse de survie par la méthode de Kaplan-Meier a été utilisée pour le critère principal composite. Le seuil de significativité était fixé à p < 0,05.

**Considérations éthiques:** l'étude a été approuvée par le comité d'éthique régional du CHU de Constantine (numéro de référence: CE/CHUC/22/09/2025) .Un consentement éclairé a été obtenu de tous les participants.

## Résultats

**Figure 1:**
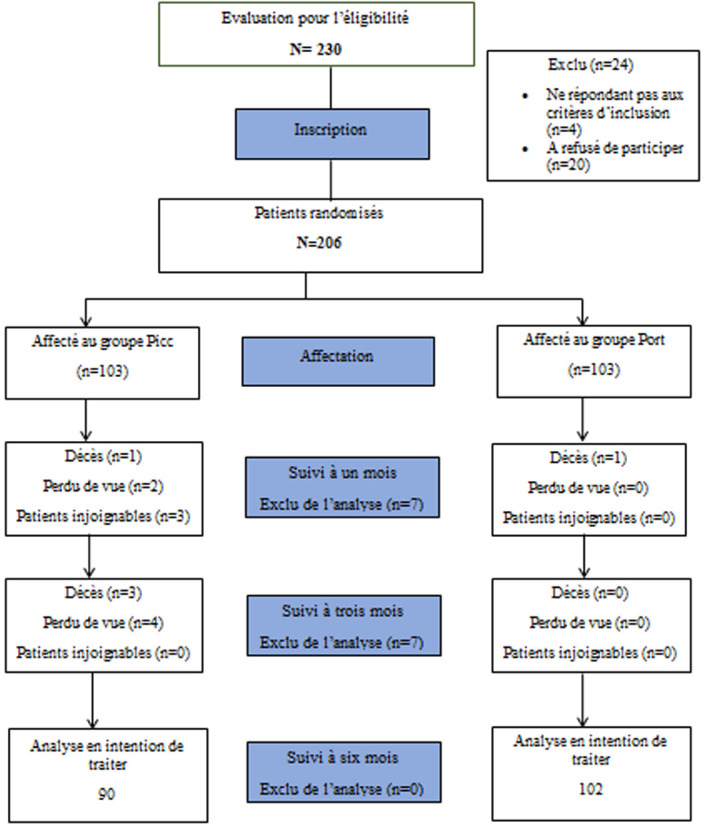
diagramme de flux du recrutement, de la randomisation, et du suivi des patients dans l'étude comparative PICC Line versus chambre implantable (n=192)

**Recrutement et caractéristiques de la population:** le recrutement des patients a été conduit entre novembre 2022 et octobre 2024. Sur les 206 patients éligibles et randomisés, 14 ont été exclus de l'analyse finale pour les raisons suivantes: cinq décès, six perdus de vue et trois injoignables (Figure 1). Ainsi, 192 patients ont été inclus dans l'analyse: 102 dans le groupe Port et 90 dans le groupe PICC.

Les caractéristiques générales étaient comparables entre les deux groupes (Tableau 1), avec un âge moyen de 57,20±12,64 ans dans le groupe Port et de 60,04±11,83 ans dans le groupe PICC, des extrêmes de 18 ans et 87 ans. Une prédominance féminine est notée dans notre ensemble de patients recrutés, un taux de 57,29% pour les femmes (n = 110) versus 42,7% pour les hommes (n = 82). Le sex-ratio homme/femme est de 0,75. Trente-neuf (20,3%) patients sont obèses.

**Tableau 1:** caractéristiques démographiques et cliniques initiales des patients à l'inclusion, par groupe de randomisation (PICC lines vs chambres implantables)

	Groupe Port	Groupe PICC	P
**Nombre de patients (n)**	102	90	**/**
**Age (ans)**	57,20±12,64	60,04±11,83	0,11
**Sexe (F/M) (%)**	57,84/ 42,15	56,66/43,33	0,87
**BMI (Kg/m^2^)**	25,91±6,14	25,85±5,51	0,94

**Incidence et types de complications:** parmi les 192 patients inclus dans l'analyse, 24 (12,5%) ont présenté une complication liée au cathéter. Les complications majeures ont concerné 21 patients (10,9%) et comprenaient des thromboses (n=7), des infections (n=7), des occlusions (n=5) et des retraits accidentels (n=2). Leur incidence était significativement plus élevée dans le groupe PICC (17,8%; 16/90) que dans le groupe Port (4,9%; 5/102), avec un risque relatif de 3,6 (IC95% 1,3-9,9; p = 0,041) (Tableau 2).

**Tableau 2:** incidence comparée des complications mineures et majeures selon le type d'accès vasculaire central

Complication (%)	Port	PICC	P
**Complications mineures**	1,04% (n=2)	0,52% (n=1)	P=0,041
**Complications majeures**	2,60% (5)	8,33% (n=16)	

**Figure 2:**
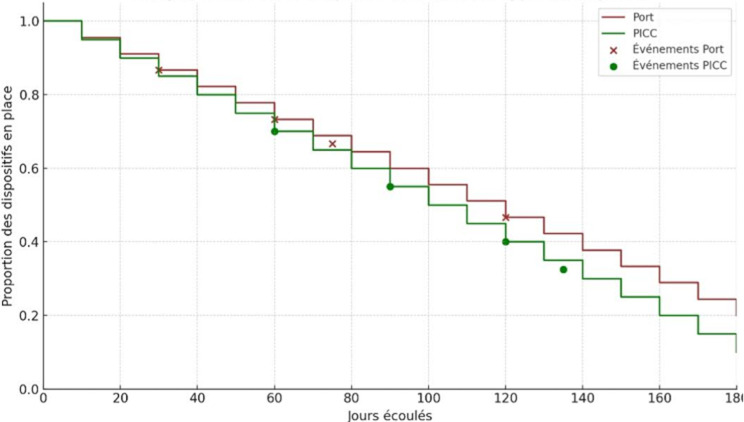
courbe de survie (Kaplan-Meier) comparant la durée de perméabilité sans complication des PICC lines et chambres implantables

**Analyse de survie sans complication:** cette différence a été confirmée par une analyse de survie de Kaplan-Meier (Figure 2) qui a montré une probabilité de survie sans complication significativement plus faible dans le groupe PICC (p<0,005). L'analyse de la temporalité a révélé que 80% de ces complications majeures sont survenues au cours des trois premiers mois suivant la pose.

**Conséquences cliniques des complications**: ces événements ont eu des conséquences cliniques directes, conduisant à l'instauration d'un traitement anticoagulant pour sept patients, à un retard de chimiothérapie pour sept autres et au remplacement du dispositif dans deux cas.

**Facteurs associés aux complications majeures:** dans l'analyse univariée, l'utilisation d'un PICC était le seul facteur significativement associé à un risque accru de complication majeure. Aucune autre variable démographique, incluant l'âge, le sexe ou l'indice de masse corporelle, n'a atteint le seuil de significativité statistique. Cependant, des tendances non significatives ont été observées, suggérant un risque plus élevé chez les patients de plus de 60 ans et ceux avec un IMC > 25 kg/m^2^ (p = 0,058 pour ces deux paramètres).

**Figure 3:**
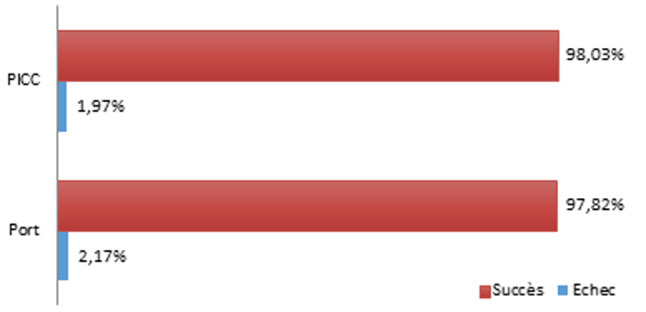
comparaison des taux de succès et d'échec d'insertion entre les chambres implantables et les PICC lines

**Résultats techniques de la pose:** le taux de réussite technique global est très élevé pour les deux dispositifs (Port: 97,8%; PICC: 98,0%), sans différence statistique significative (p > 0,05) (Figure 3). Concernant le succès de la ponction en une seule tentative, le taux était de 95,6% pour les Ports et de 90,0% pour les PICC. Bien que numériquement en faveur des Ports, cette différence n'était pas statistiquement significative lorsqu'on comparait directement ces deux dispositifs (p > 0,05). La durée moyenne de l'intervention était significativement plus longue pour la pose d'un Port (32,17 ± 6,35 minutes) que pour celle d'un PICC (18,68 ± 6,03 minutes) (p=0,001).

**Analyse économique:** dans notre cohorte, l'analyse du coût global révèle une différence substantielle entre les deux dispositifs, avec un coût moyen total de 160123,41±27633,60 DA pour les Ports et de 69±5105,40 DA pour les PICCs (Tableau 3).

**Tableau 3:** comparaison des coûts moyens (en dinar algérien, DA) associés à la pose, l'entretien, la gestion des complications et le retrait entre les chambres implantables et les PICC lines

Groupe/Les coûts (DA)	Port N= 102	PICC N= 90	P
**Coût moyen de pose ± écart-type**	154344,13±0,00	36184,00±0,00	/
**Coût moyen d'entretien±écart-type**	00,00±00,00	5128,29±1548,28	/
**Coût moyen des complications ± écart-type**	916,42±3156,85	1168,44±3524,96	0,54
**Coût moyen de retrait ± écart-type**	5321,07±2646,82	1168,44±3524,96	< 0,001
**Coût moyen total±écart-type**	160123,41±27633,60	43259,69±5105,40	< 0,001

**Evaluation de la qualité de vie:** l'outil d'évaluation de la qualité de vie est le questionnaire EORTC QLQ-C3, 192 patients ont été recrutés sur une période de vingt-quatre mois (groupe PICC n = 90; groupe Port, n = 102). Les taux globaux de réponse aux enquêtes pour les mois 1 et 3 étaient respectivement de 71% et 52%. À un mois: aucune différence significative n'a été notée entre les deux groupes pour les scores de qualité de vie EORTC 30. À trois mois: les scores de santé globale étaient de 61,66±16,48 pour le groupe PICC et de 65,16±18,29 pour le groupe Port (P <0,09). Le score de douleur était de 56,18±23,89 pour les PICC et de 50,35±22,99 pour les Port (p < 0,07). Le score de la capacité fonctionnelle était de 73,4±14,03 pour les Port et de, 7±12,1 pour les PICC (p=0,91). Le score pour l'effet négatif du dispositif sur le bien-être psychosocial était de 92±22,26 pour les PICC et de 54,87±25,69 pour les Ports (p=0,87) (Tableau 4).

**Tableau 4:** scores moyens de la qualité de vie (avec écarts-types) selon les domaines de l'EORTC QLQ-C30, comparant les patients porteurs de PICC lines et de chambres implantables à 1 mois et 3 mois après la pose

	1 mois		P	3 mois		P
	PICC	PORT		PICC	PORT	
**Santé globale**	60,13±17,66	61,72±13,73	0,68	61,66±16,48	65,16±18,29	0,09
**Capacité fonctionnelle**	65,92±6,53	62,49±10,12	0,53	73,7±12,1	73,4±14,03	0,91
**Capacité à accomplir toute forme de travail et d’activité de loisir**	41,31±7,75	44,37±12,94	0,18	66,95±5,79	69,17±15,01	0,19
**Etat émotionnel**	51,54±16,78	54,39±16,22	0,36	59,05±18,14	65,63±16,04	0,055
**Capacité cognitive**	67,10±20,91	62,01±19,70	0,079	89,67±25,70	83,21±22,53	0,064
**Capacité à maintenir les relations sociales**	38,74±12,61	35,95±19,98	0,65	55,92±22,26	54,87±25,69	0,87
**Fatigue**	43,14±23,69	40,51±20,30	0,24	44,73±19,41	47,62±22,53	0,20
**Nausées et vomissements**	19,19±14,63	17,14±15,29	0,49	9,74±5,15	7,72±8,34	0,47
**Douleur**	53,59±22,19	55,82±24,53	0 ,61	56,18±23,89	50,35±25,99	0,07
**Dyspnée**	73,20±17,66	68,25±24 ,99	0,21	53,98±19,27	60,85±28,42	0,060
**Insomnie**	59,48±18,03	60,32±26,68	0,84	46,41±16,44	53 ,56±26,10	0,32
**Manque d'appétit**	51,63±22,42	57,14±26,39	0,23	65,36±18,81	48,15±22,22	<0,001
**Constipation**	18,30±26,09	24,39±23,60	0,24	6,12±17,58	10,42±17,84	0,291
**Diarrhée**	34,33±17,82	22,92±21,91	0,16	43,06±30,72	39,02±26,37	0,09
**Difficultés financières**	39,87±26,68	48,93±21,52	0,053	62,75±26,37	67,58±23,00	0,39

## Discussion

Le choix du dispositif d'accès veineux central optimal pour l'administration de la chimiothérapie repose sur une évaluation équilibrée entre la sécurité, la faisabilité technique, le coût et l'impact sur la qualité de vie des patients. Notre étude prospective, randomisée et monocentrique, menée dans un contexte algérien, a comparé les chambres à cathéter implantables (Ports) et les cathéters centraux insérés par voie périphérique (PICC). Les résultats démontrent que les Ports sont associés à un risque significativement plus faible de complications majeures, mais à un coût initial plus élevé, sans différence significative sur la qualité de vie globale mesurée par un questionnaire générique [10].

Le principal critère de sécurité, l'incidence des complications majeures, était significativement plus faible avec les Ports (4,9%) qu'avec les PICC (17,8%), correspondant à un risque relatif de 3,6. Ce résultat conforte les données de la littérature internationale, où les PICC sont régulièrement associés à un risque accru de complications thrombotiques et infectieuses [8,9]. La majorité de ces événements dans notre cohorte est survenue précocement, dans les trois premiers mois suivant la pose, soulignant une période critique justifiant une surveillance renforcée, en particulier pour les PICC [8].

Sur le plan technique, les deux dispositifs ont montré une fiabilité de pose très élevée et comparable (>97%), ce qui est conforme aux standards attendus avec l'utilisation systématique de l'échographie [11]. Cependant, la durée procédurale était significativement plus courte pour la pose des PICC, reflétant leur caractère moins invasif. Cet avantage pratique doit être nuancé par la nécessité, pour les Ports, d'une implantation dans des conditions opératoires plus strictes, pouvant représenter une contrainte logistique dans certains établissements [12].

L'analyse médico-économique a mis en évidence une différence majeure : pour une chimiothérapie de durée inférieure à six mois, le coût total moyen des Ports était environ quatre fois supérieur à celui des PICC. Ce surcoût, principalement lié à l'acte d'implantation, est un élément décisif dans des environnements aux ressources limitées [12]. Néanmoins, une évaluation complète devrait intégrer le fardeau économique indirect des complications plus fréquentes associées aux PICC, bien que celui-ci n'ait pas atteint la significativité statistique dans notre analyse des coûts directs [9,11]. Contrairement à nos attentes, l'évaluation de la qualité de vie à l'aide du questionnaire EORTC QLQ-C30 n'a pas révélé de différence significative entre les groupes, en dehors de tendances modestes. Ce résultat suggère que l'impact global de la maladie et du traitement pourrait masquer des différences plus spécifiques liées au dispositif, ou qu'un outil de mesure générique n'est pas suffisamment sensible pour les détecter [13].

Les principales forces de cette étude sont son design prospectif randomisé et l'évaluation multidimensionnelle des dispositifs. Elle fournit des données factuelles précieuses pour un contexte de soins aux ressources contraintes. Ses limites incluent son cadre monocentrique, une puissance statistique limitée pour certains événements, et une population hétérogène en termes de types tumoraux.

## Conclusion

Nos résultats, cohérents avec les données de la littérature internationale, suggèrent que le choix entre Port et PICC doit reposer sur une décision clinique personnalisée. Un schéma décisionnel pratique pourrait émerger de cette synthèse: le Port pourrait être privilégié chez les patients nécessitant une chimiothérapie de longue durée (> 6 mois) ou présentant des facteurs de risque thrombotiques, maximisant ainsi son avantage en matière de sécurité. À l'inverse, le PICC pourrait représenter une option valable pour des traitements de plus courte durée chez des patients à faible risque de complications, notamment dans des contextes où les contraintes économiques ou d'accès au bloc opératoire sont prégnantes. Des études prospectives avec une analyse coût-utilité robuste intégrant le fardeau des complications sont nécessaires pour guider des recommandations formelles dans des contextes économiques variés.

### Etat des connaissances sur le sujet

- La prise en charge des cancers nécessite un accès veineux central fiable pour l'administration prolongée de chimiothérapie (les chambres implantables et les cathéters centraux insérés par voie périphérique sont les deux dispositifs les plus utilisés);
- Les données internationales établissent un compromis entre sécurité et coût: les Ports sont associés à un risque plus faible de complications majeures (thrombotiques, infectieuses) mais génèrent un coût initial supérieur à celui des PICC;
- En Afrique, et notamment en Afrique du Nord, les données comparatives prospectives manquent pour guider les décideurs et les cliniciens dans le choix du dispositif le plus adapté aux réalités économiques et organisationnelles des systèmes de santé locaux.

### Contribution de notre étude à la connaissance

- La première étude prospective randomisée menée en Algérie, à comparer directement la sécurité, le coût et l'impact sur la qualité de vie des Ports et des PICC en oncologie;
- Elle fournit des données factuelles robustes, issues d'un contexte africain, sur le compromis sécurité-coût entre les deux dispositifs;
- Et elle apporte des éléments concrets pour guider les décisions cliniques dans des contextes aux ressources limitées.
